# Initial Institutional Experience and Workflow of Biology-Guided Radiotherapy (BgRT) for a Patient With Non-small Cell Lung Cancer

**DOI:** 10.7759/cureus.96513

**Published:** 2025-11-10

**Authors:** Young Suk Kwon, Bin Cai, Chenyang Shen, Thomas I Banks, Rameshwar Prasad, Orhan K Oz, Tu Dan, Aurelie Garant, Kenneth Westover, David Sher, Robert Timmerman, Shahed Badiyan

**Affiliations:** 1 Radiation Oncology, University of Texas, Southwestern Medical Center, Dallas, USA; 2 Radiology, University of Texas, Southwestern Medical Center, Dallas, USA

**Keywords:** biology-guided radiotherapy (bgrt), fdg, linac-based, pet, scintix

## Abstract

We describe a case of a 69-year-old gentleman with a biopsy-proven non-small cell lung cancer (NSCLC) who was treated with the SCINTIX biology-guided radiotherapy (BgRT). In this report, we focus on the development of our institutional clinical workflow for BgRT to ensure a safe and seamless process. This entails the initial import of diagnostic PET/CT with fluorodeoxyglucose-avid lesion(s), 4D CT simulation, contouring including the biology tracking zone, a patient modeling session for planning PET, and radiation treatment (RT). BgRT is a promising technology that has the potential to allow functional adaptation of RT. In our patient with NSCLC who was treated with 5-fraction BgRT, we were able to achieve the threshold for activity concentration and a sufficiently normalized tumor signal. Furthermore, the time analyses of treatment steps demonstrated an efficient workflow.

## Introduction

Biology-guided radiotherapy (BgRT) is a novel technology for delivering stereotactic radiotherapy. BgRT therapy leverages the PET avidity of the target tumor, eliminating the need for traditional motion management. Using PET signals for guidance ensures highly conformal treatment with real-time target tracking. Currently, the only platform offering BgRT therapy is the RefleXion X1 PET-LINAC system (RefleXion Medical, Hayward, CA) [1]. The X1 system is a compact radiation treatment platform that integrates a ring gantry LINAC with a PET/CT scanner. It features a 6 MV FFF LINAC with a dose rate of 1000 MU/min at an 85 cm source-to-axis distance, mounted on a rotating O-ring gantry capable of rotating at 60 RPM. Two PET detector arrays are positioned orthogonally to the MV beamline. The system is also equipped with a binary multi-leaf collimator featuring 2 cm or 1 cm jaw sizes, as well as a six-degree-of-freedom couch for precise axial radiation delivery.

A clinical trial conducted under an Investigational Device Exemption (IDE) demonstrated the safety and efficacy of BgRT therapy for treating primary or metastatic bone and lung lesions, leading to its FDA approval for these specific indications [2]. Understandably, the adoption of this technology requires multidisciplinary effort from physicians, physicists, radiation therapists, and nuclear medicine technologists. Herein, we describe our institutional workflow and initial experience of BgRT in treating a patient with early-stage non-small cell lung cancer (NSCLC).

## Technical report

Patient inclusion criteria

Patients with a lung or bone tumor with gross tumor volume (GTV) >1 cm but <5 cm and planned for treatment with five fractions or less and a dose of ≥8 Gy per fraction were selected for our initial cases. A minimum SUV max requirement of 6.0 was established based on the results of the IDE study [2]. Lastly, cases with targets adjacent to organs-at-risk (OAR) structures with high SUV uptake were deemed unsuitable for BgRT.

Institutional workflow

**Figure 1:**
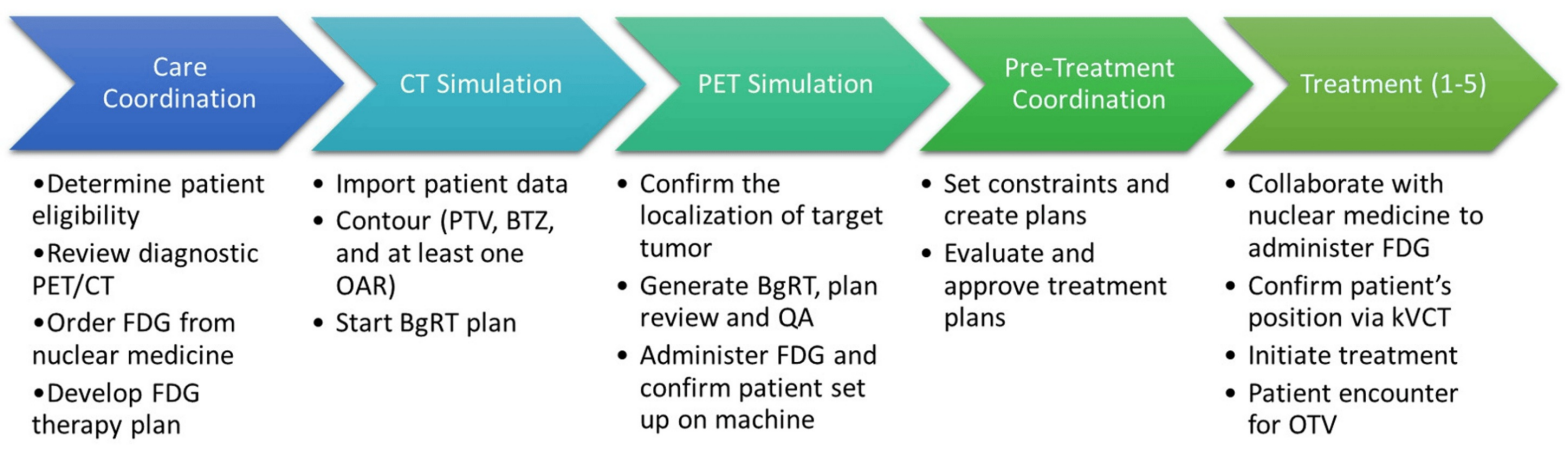
BgRT institutional workflow. BgRT: biology-guided radiotherapy, FDG: fluorodeoxyglucose, PET/CT: positron emission tomography/computed tomography, PTV: planning target volume, BTZ: biology tracking zone, OAR: organ-at-risk, kVCT: kilovoltage computed tomography, OTV: on-treatment visit

A detailed process map for the clinical workflow of the BgRT machine had been described previously [3]. We implemented several modifications to accommodate our institutional circumstances (Figure 1). The hallmark of our workflow was close communication among all parties involved. First, eligible cases were evaluated jointly by the patient’s radiation oncologist and medical physicists during the initial consultation. Once an appropriate patient was selected, the nuclear medicine department was contacted to order the fluorodeoxyglucose (FDG) doses and coordinate FDG injections for both the PET modeling session and the treatment sessions. A standard 4D CT simulation was performed, and target volumes, the biological tracking zone (BTZ), a 3D volume surrounding the PET-avid tumor, and OARs were contoured in Eclipse (Varian Medical Systems, Palo Alto, CA, USA).

These contours were imported into the RefleXion treatment planning system (TPS) for the patient’s PET modeling session. During the modeling session on the X1 system, PET images were acquired following FDG injection. These PET images were fused with CT images in the RefleXion TPS, and both the physician and physicist confirmed the presence of the tumor/target. The planning phase is selected based on the longest dwell time used for GTV contouring, with the average CT phase applied for planning and dose calculation. The BgRT plan was then generated based on the PET images from the X1 machine, departmental treatment guidelines, dosimetric constraints, and the supervising physician’s intent. A backup image-guided radiotherapy (IgRT) plan is routinely generated when BgRT plans fail to meet pre-scan criteria for activity concentration (AC), normalized target signal (NTS), or gamma analysis.

The physician reviewed the BgRT plan and approved the quality assurance provided by the medical physics team. Before each subsequent treatment fraction, the patient was injected with FDG, and a 45‑minute uptake period was allowed. After patient setup, positioning was confirmed with kilovoltage computed tomography. A short-duration PET scan was obtained to assess the reliability and stability of the PET signals. AC, NTS, and gamma analysis were performed to ensure the stability of the PET signal on the treatment day. In BgRT, AC refers to the absolute PET signal intensity within the tumor and must be greater than 5 kBq/mL. At the same time, NTS represents the ratio of tumor signal to background activity and must be greater than 2 to ensure reliable tracking. Throughout treatment delivery, the system continuously monitored and analyzed real-time signals, making necessary adjustments to the beam firing positions to ensure accurate dose delivery. A medical physicist conducted a post-treatment radiation survey to ensure that the treatment room and devices were not contaminated.

Case presentation

The patient was a 69-year-old man with chronic obstructive pulmonary disease (COPD) requiring 2-3 liters of supplemental oxygen who was found to have a pulmonary nodule on annual screening CT. Imaging demonstrated a spiculated nodule in the lingula measuring 2.8 cm in the setting of moderate emphysema. Transbronchial fine-needle aspiration of the left upper lobe lesion revealed adenocarcinoma, and mediastinal nodes at levels 4 L and 7 were negative. The patient received 5500 cGy in five fractions over 12 days with SCINTIX BgRT without complications. At 20 months of follow-up, there was no evidence of recurrent disease on clinical or radiographic evaluation.

Planning and dosimetry

Contouring

A phase with the longest dwell time was selected for contouring to approximate the average PET location. BgRT planning target volume (PTV) was defined as a single-phase GTV with a 5 mm uniform margin. The BTZ was defined as a 10 mm expansion of the internal target volume (ITV) (contoured on maximum intensity projection) obtained from the 4D CT images. The average CT image was used for plan and dose calculation. The BgRT PTV was 63.27 cc, significantly smaller than the ITV-based IgRT PTV of 81.57 cc.

Dose Volume Histogram

**Figure 2:**
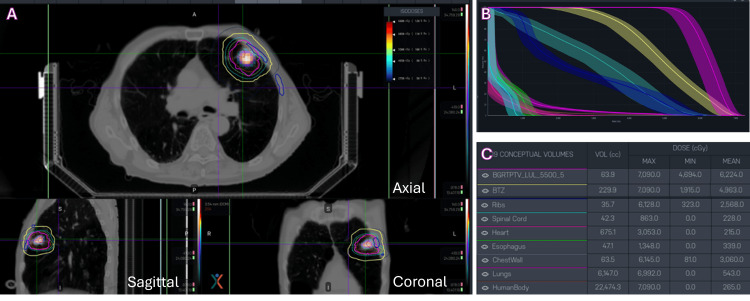
Radiation treatment plan for the case patient: (A) contouring in axial view (top panel), sagittal view (bottom left panel), and coronal view (bottom right panel); (B) bDVH (top right panel); and (C) dose volume characteristics (top bottom panel). bDVH: bounded dose-volume histogram

The BgRT for SCINTIX treatment and the traditional IgRT plans with the ITV approach were created for comparison (Figure 2A-2C). A bounded dose-volume histogram (bDVH) was created as the BgRT TPS simulates multiple DVHs using possible permutations, and bDVH visually represents these variations with confidence intervals (Figure 2C) [1].

**Figure 3:**
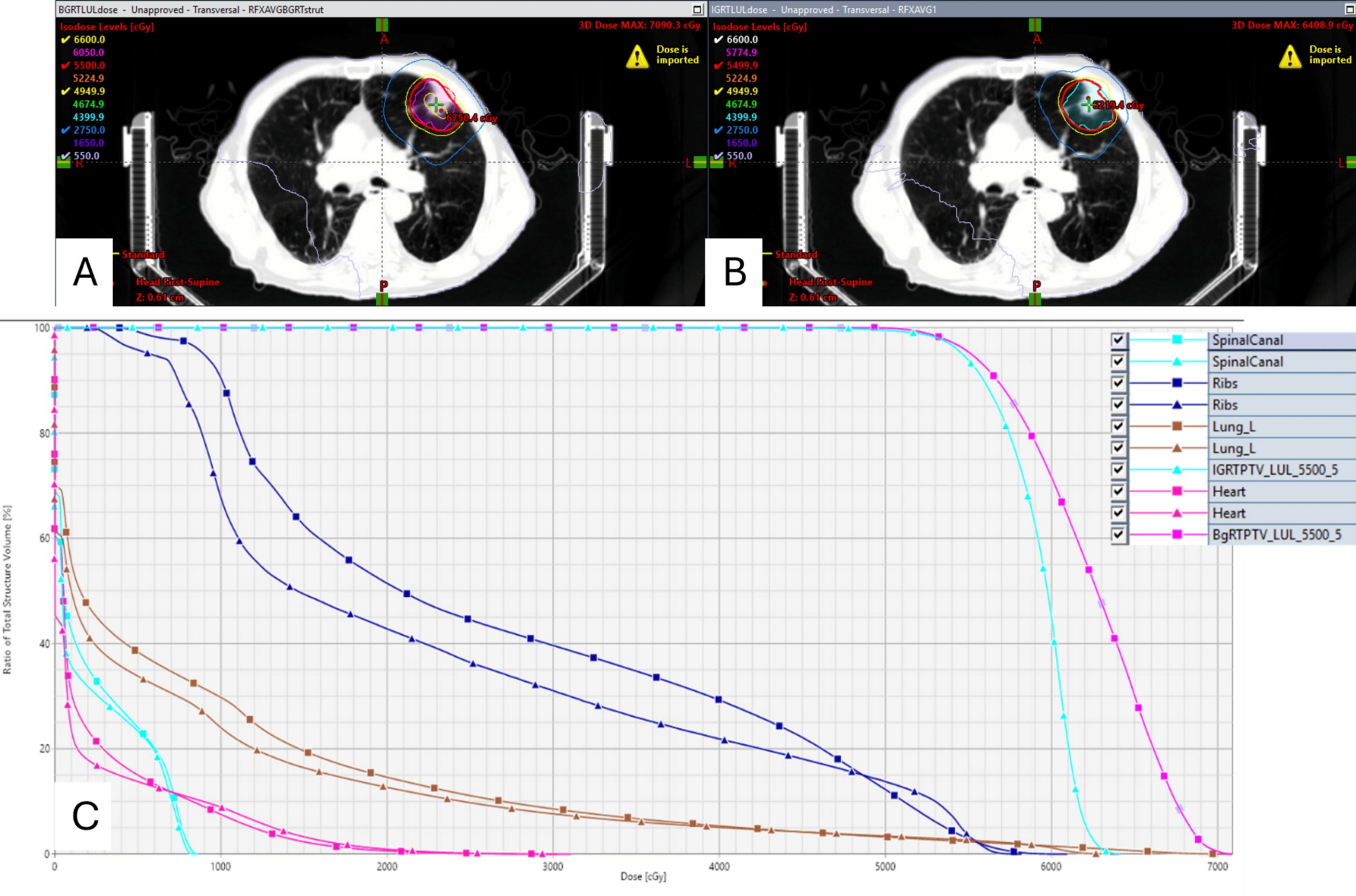
Dose distribution comparison between (A) BgRT (top left panel) and (B) conventional IgRT (top right panel). (C) Dose volume comparison between BgRT and IgRT (bottom panel). In the DVH curves, all square markers correspond to BgRT, and all triangle markers correspond to conventional IgRT. The target volumes are represented by the BgRT PTV in pink squares and the IgRT PTV in blue triangles. OARs include the spinal canal (cyan), ribs (brown), left lung (dark blue), and heart (magenta), which are shown as squares for BgRT and triangles for IgRT. BgRT: biology-guided radiotherapy, IgRT: image-guided radiotherapy, DVH: dose-volume histogram, PTV: planning target volume, OARs: organs-at-risk

When compared to the traditional IgRT plan, the BgRT plan had slightly better PTV coverage (95.4% vs 93.8%) (Figure 3A-3C). The BgRT plan was hotter compared to the IgRT plan. The maximum, mean, and minimum doses of the BgRT PTV were 128.9%, 113.4%, and 86.8%, respectively, compared to the IgRT PTV doses of 116%, 107.6%, and 74.0%. V20 of the left lung was comparable between the BgRT and IgRT (14.5% vs 12.7%). The mean heart doses of the BgRT and IgRT plans were 3.9 and 3.7%, respectively (Figure 3C). Chest wall metrics, such as V30 (<30 Gy), were not evaluated in this analysis.

Treatment Delivery

**Figure 4:**
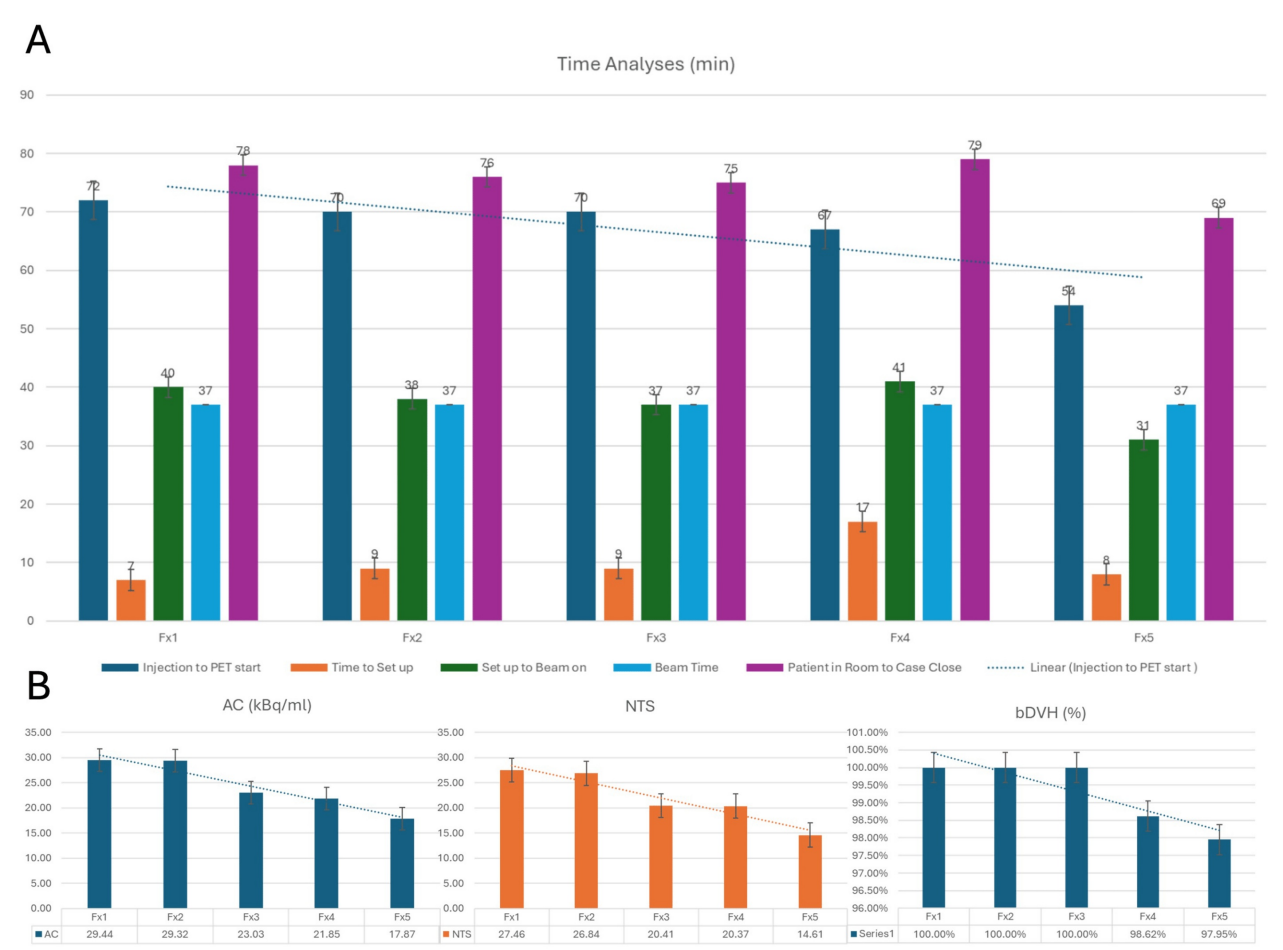
(A) Time analyses over five fractions (injection to PET start; time to set up; setup to beam on; beam time; and patient in room to case close). (B) The change in AC, NTS, and percent bDVH over five fractions. PET: positron emission tomography, AC: activity concentration, NTS: normalized target signal, bDVH: bounded dose-volume histogram

The above patient was able to complete the treatment safely without treatment breaks. The beam time was 37 minutes for each fraction. The BgRT plan deliverability was first assessed by evaluating the AC and NTS on converted PET images to meet the NTS >2, an absolute hard constraint, and the AC >5 kBq/mL, a preferable goal. The changes in AC and NTS values over time demonstrated a decreasing trend (Figure 4).

## Discussion

Our index patient case demonstrates the feasibility of BgRT for early-stage NSCLC. We described our institutional experience in developing a clinical workflow and emphasized a few important practical considerations when utilizing the SCINTIX X1 machine. In our collaborative team atmosphere with a streamlined workflow, treatment delivery was consistently under 80 minutes, with a mean beam time of 37 minutes, while meeting AC and NTS thresholds for deliverable BgRT.

To the best of our knowledge, this is among the first published reports of a patient treated with BgRT. This patient had limited pulmonary function due to COPD. Moreover, BgRT obviated the need for deep inspiratory breath-hold or active breathing control, which are often challenging or not feasible in patients with compromised pulmonary function. While BgRT involves a longer setup and delivery time, it uniquely enables precise, motion-compensated treatment in such patients, highlighting its clinical feasibility and potential applicability beyond conventional IgRT. The RefleXion X1 enables real-time tracking of tumor location, accounting for interfractional variations to enhance precision. In a dosimetric comparison study, Seyedin et al. evaluated the radiation exposure to the total lung and chest wall for the treatment of an upper lobe tumor when treated with BgRT. It revealed numerically lower V20 (7.5%) compared to VMAT (9.3%) and IMPT (10.8%) [4]. Similarly, V20 for our patient was 14.5%, which is within the constraint limit of 10-15% [5]. When compared to the lung V20 achieved with BgRT, the V20 from the IgRT backup plan created in Varian Eclipse was lower (15.0% vs 12.7%). On the other hand, the mean PTV coverage of BgRT was slightly higher than that of IgRT (95.4% vs 93.8%). While BgRT appears to provide effective radiation treatment based on follow-up CT scans at 20 months, further work is required to demonstrate the benefits of BgRT over conventional IgRT techniques.

The cornerstone of successful BgRT relies on building an effective team composed of physicians, medical physicists, dosimetrists, and radiation therapists. At our institution, we currently have one physician, one medical physicist, one nuclear medicine technologist, and two radiation therapists assigned to the RefleXion X1 workflow (Figure 1). From discussions on patient eligibility to simulation, treatment planning, and treatment, all team members are included in communications via an institutional messaging system to streamline planning and delivery. Full treatment fractions are scheduled only after confirming BgRT eligibility, with the planning phase based on the longest dwell time for GTV contouring. For each BgRT treatment plan, we also routinely create a backup IgRT plan if the BgRT plan does not meet the pre-scan criteria on AC, NTS, and gamma analyses [6].

Based on our time analyses, a decreasing trend in time was observed between the injection of the radioactive substance and the start of the PET/CT (Figure 3). Reducing this preparatory time can increase the overall efficiency of the workflow. The beam time was consistently 37 minutes throughout the 5-fraction treatment. We anticipate that the steps involved in the BgRT workflow will become more streamlined with experience. Ultimately, we aim to consistently achieve a 60-minute time window for FDG injection to PET/CT pre-scan and for treatment plan approval to completion of treatment.

The BgRT plan's feasibility is closely related to AC and NTS. In our five-fraction BgRT for NSCLC, we were able to monitor these values and observed a decreasing trend (Figure 4). More data are required to determine if decreasing values are indicative of a favorable treatment response. Our findings are consistent with previously published work, where normalized AC values trended downward from the first to the last fraction (p < 0.05) [7]. Finally, the BgRT system utilizes bDVH, which encompasses multiple possible DVH visually describing the best to the worst dosimetric variations [1]. The interfractional percent reduction of bDVH is negligible in our patient case, highlighting minimal dosimetric variations.

There are a few limitations of BgRT. First, the eligibility criteria, including the minimum SUV threshold of the target, tumor size, and FDG uptake from surrounding OARs, make it challenging for the routine adoption of this technology for all tumors. Given the complex workflow, one may argue against the use of BgRT for treating patients with NSCLC, considering the favorable outcomes of conventional stereotactic ablative body radiation (SAbR). In addition, the average BgRT treatment delivery time was approximately 80 minutes, including beam-on and setup, compared with reported treatment times of 20-45 minutes for conventional SAbR using image guidance without breath-hold techniques [8]. As we await a more seamless workflow and improvements in hardware, such as a PET detector and treatment planning optimizer, we hope to address some of the challenges of BgRT.

## Conclusions

Our study demonstrated the feasibility of BgRT through a case example of our patient with NSCLC. Additionally, we have highlighted the collaborative, multidisciplinary team effort that developed an efficient and effective workflow. Lastly, the utilization of interfractional time analyses and BgRT-specific parameters, including AC, NTS, and bDVH, demonstrated potential for improvement and consistently confirmed acceptable treatment delivery from the initial to the last fractions. Further work is required to compare short- and long-term oncologic and toxicity outcomes between the conventional SAbR and BgRT.
